# Case Report: Steroid-responsive immune-mediated thyroiditis in a young dog with multi-systemic pyogranulomatous inflammation

**DOI:** 10.3389/fvets.2025.1662178

**Published:** 2025-12-15

**Authors:** Kyle L. Granger, Claire D. Tucker, Amanda Garrick, Paula Yoon, Christopher Olmo, A. R. Moore, Paula A. Schaffer, Sarah Shropshire, Kelly E. Hall

**Affiliations:** 1 Department of Clinical Sciences, College of Veterinary Medicine and Biomedical Sciences, Colorado State University, Fort Collins, CO, United States; 2 Department of Environmental and Radiological Health Sciences, Colorado State University, Fort Collins, CO, United States; 3 Department of Microbiology, Immunology, and Pathology, College of Veterinary Medicine and Biomedical Science, Colorado State University, Fort Collins, CO, United States

**Keywords:** immune-mediated disease, canine hypothyroidism, thyroiditis, dog, steroid-responsive disease

## Abstract

A 3-year-old neutered male Posavac Hound presented with rapidly progressive cervical swelling, respiratory distress, and systemic inflammation. Diagnostics confirmed severe hypothyroidism [total thyroxine (TT4) < 0.5 μg/dL, thyroid-stimulating hormone (TSH) 5.54 ng/dL], elevated thyroglobulin autoantibodies (TgAA 131%), hyperglobulinemia, hypoalbuminemia, and mild ionized hypercalcemia. Imaging showed bilateral thyroid enlargement with cavitated necrosis, pulmonary nodules, and lymphadenopathy. Cytology revealed pyogranulomatous inflammation without infectious organisms, and infectious disease screening was negative. Protein electrophoresis and immunofixation revealed elevated IgG4, suggesting immune-mediated dysregulation. The dog responded rapidly to prednisolone and thyroid hormone supplementation, with resolution of clinical signs and normalization of inflammatory markers by ~60 days. This report describes a unique case of immune-mediated thyroiditis with systemic inflammatory involvement that responded to steroids, emphasizing the importance of considering immune-mediated thyroiditis as a differential diagnosis for acute cervical neck swelling in a young dog with concurrent pulmonary involvement.

## Background

Thyroiditis is an inflammation of the thyroid gland that can be caused by autoimmune processes, infection, trauma, drug exposure, or neoplastic infiltration in both humans and dogs (1–7). In people, thyroiditis encompasses several distinct entities: Hashimoto’s disease, acute or subacute (de Quervain’s) thyroiditis, and Riedel’s thyroiditis, each defined by clinical features such as painful or painless neck swelling, systemic malaise, and variable thyroid hormone profiles (1–7). Some people experience transient thyrotoxicosis from follicular disruption, whereas others progress directly to hypothyroidism as chronic inflammation or fibrosis destroys functional tissue (3–5, 7).

Given the absence of a well-described canine model for acute, fulminant thyroiditis, human medical literature provides an essential framework for understanding potential pathogenic mechanisms. In people, thyroiditis encompasses several distinct entities. Hashimoto’s disease is characterized by autoantibodies against thyroglobulin and thyroid peroxidase, whereas Riedel’s thyroiditis and the emerging IgG4-related subtype feature invasive fibrosis that can extend into perithyroidal soft tissue, lung, or other organs (5, 7–10). IgG4-related disease in humans involves infiltration of IgG4-positive plasma cells, leading to fibrotic inflammatory lesions in multiple organs, including the thyroid, and is notable for its aggressive tissue invasion and steroid responsiveness (11, 12).

In dogs, the most recognized immune-mediated thyroid disorder is chronic lymphocytic thyroiditis, a slowly progressive analog of human Hashimoto’s disease that culminates in glandular atrophy and overt hypothyroidism without systemic inflammation (13–18). Clinical signs typically include lethargy, weight gain, dermatologic changes, cold intolerance, bradycardia, neuromuscular weakness, reproductive disturbances, behavioral alterations, and occasionally mild anemia. Although toxin-associated acute thyroiditis has been described in connection with *Toxoplasma gondii* infection (19), an acute, steroid-responsive, immune-mediated form has not been characterized in the veterinary literature. For clarity, we proposed the provisional term steroid-responsive immune-mediated thyroiditis (SRIMT) to describe a case of sudden cervical swelling, profound hypothyroidism, and multi-organ pyogranulomatous inflammation that resolved rapidly with immunomodulatory steroid therapy and recognized a need for further characterization of this process in dogs.

This report documents an SRIMT in a young dog. We detailed its clinical presentation, diagnostic evaluation, and therapeutic response and compared this syndrome with established thyroiditis phenotypes in both human and veterinary medicine. It is important to differentiate this presentation from more common canine diseases causing acute cervical swelling and systemic illness, such as deep cervical abscessation, migrating foreign bodies, sialoceles, or neoplasia (e.g., lymphoma or thyroid carcinoma). This case was unique due to its bilateral thyroidal origin, concurrent systemic pyogranulomatous lesions, and rapid response to immunomodulation rather than antimicrobial or surgical intervention.

## Case presentation

A 3-year-old neutered male Posavac Hound (36.4 kg) presented to a referral veterinarian with 3 days of inappetence, drooling, progressive neck swelling, and acute dysphonia. The dog was tachycardic with respiratory stridor. Initial diagnostics revealed a low total thyroxine (TT4: <0.5 μg/dL). Radiographs showed ventral cervical soft tissue swelling, a ~ 5 cm caudal lung mass, and a bronchointerstitial pattern. Cytology of a neck aspirate indicated pyogranulomatous inflammation; bacterial and fungal cultures of neck and lung aspirates were negative. The dog was hospitalized for supportive care and broad-spectrum antibiotics (ampicillin/sulbactam and enrofloxacin), but was referred to a veterinary teaching hospital (VTH) the next day due to concerns regarding neoplasia or an atypical infectious process.

### Clinical presentation to VTH

Upon presentation to the VTH, the dog was hemodynamically stable (HR 96 bpm, strong bilateral femoral pulses) with a rectal temperature of 36.6 °C (97.8 °F). A firm, non-painful ventral cervical swelling extended bilaterally from the caudal mandibular ramus to the thoracic inlet, laterally spanning the full width of the neck. The mass adhered to the underlying tissues and obscured normal cervical landmarks, including the trachea. There was scant serosanguinous to hemorrhagic discharge from the left nostril and mild conjunctival hyperemia oculus uterque. On admission, blood tests revealed hyperglobulinemia, hypercholesterolemia, mild hypercapnia, and ionized hypercalcemia (Tables 1, 2). An IDEXX canine SNAP 4Dx® test was negative for *Dirofilaria immitis antigen and Anaplasma phagocytophilum/ platys, Borrelia burgdorferi, and Ehrlichia canis/ewingii* antibodies, helping to rule out these common vector-borne infectious etiologies as a cause of the systemic inflammation and lymphadenopathy. A repeat neck ultrasound confirmed bilateral, moderately heterogeneous, mildly hyperechoic thyroid masses (right 2.18 × 2.66 cm; left 2.4 × 2.7 cm) and mandibular and medial retropharyngeal lymphadenopathy (right 1.5 cm; left 2.7 cm, Supplementary Figure 1). The surrounding perithyroidal and subcutaneous tissues were diffusely thickened, hyperechoic, and edematous, with indistinct fascial planes, which is consistent with severe regional cellulitis and edema (Supplementary Figure 1). Ultrasound-guided FNAs of the thyroid masses and mandibular nodes revealed mild suppurative inflammation with necrotic cellular debris, but no evidence of infectious agents, and could not confirm the sampling of thyroid tissue. Bacterial, fungal, and mycobacterial cultures of lymph node samples; whole-blood polymerase chain reaction (PCR) for *Francisella tularensis* and *Yersinia pestis*; and immunofluorescence assays for *Leishmania infantum* were all negative. The dog was hospitalized overnight on maropitant (1 mg/kg IV q24h), ondansetron (0.5 mg/kg IV q8h), and a Lactated Ringer’s CRI (1.5 mL/kg/h).

**Table 1:** Biochemistry panels in chronological order of a 3-year-old Posavac Hound with pyogranulomatous immune-mediated thyroiditis.

Test	Day 1	Day 4	Day 31	Day 235	RI	Units
A/G	0.59 (L)	0.86 (L)	1.74	2.18	0.9–2.4	Ratio
ALB	20.0 (L)	30	40	37	30–43	g/L
ALP	77	95	850.0 (H)	250.0 (H)	15–140	IU/L
ALT	39	34	359.0 (H)	40	10–90	IU/L
AN GAP	15	19	26.0 (H)	21	12–23	mmol/L
AST	103.0 (H)	28	41	15	15–45	IU/L
BUN	4.64	10	4.28	2.14 (L)	2.5–10.7	mmol/L
CA	2.4	2.6	2.6	2.55	2.25–2.87	mmol/L
CHOL	8.05 (H)	8.52 (H)	6.37	4.3	3.37–7.77	mmol/L
CK	743.0 (H)	248	385.0 (H)	215	50–275	IU/L
CL	117.3	104.1 (L)	107.0 (L)	113.9	108–118	mmol/L
CREAT	82.21	90.17	50.39 (L)	55.69	53–141	μmol/L
GGT	0	1	27.0 (H)	5	0–9	IU/L
GLOB	34.0 (H)	35.0 (H)	23	17	15–32	g/L
GLU	6.72 (H)	5.72	5	5.88	3.9–6.4	mmol/L
HCO3-	19.5	26.8 (H)	22.5	19.1	15–25	mmol/L
IRON	5.73 (L)	17.54	64.98 (H)	18.97	14.3–48.3	μmol/L
K	3.76 (L)	5.16	4.48	4.77	3.9–5.4	mmol/L
MG	0.7 (L)	0.82	0.78	0.95	0.74–0.99	mmol/L
NA	148	145	151	149	142–152	mmol/L
PHOS	0.97	1.91	1.61	2.07 (H)	0.81–1.93	mmol/L
T-BIL	3.42	1.71	1.71	0	0–3.4	μmol/L
TP	54	65	63	54	50–70	g/L

A/G, albumin/globulin ratio, ALB, albumin; ALP, alkaline phosphatase; ALT, alanine aminotransferase; AN GAP, anion gap; AST, aspartate aminotransferase; BUN, blood urea nitrogen; CA, calcium; CHOL, cholesterol; CK, creatine kinase; CL, chloride; CREAT, creatinine; GGT, gamma-glutamyl transferase; GLOB, globulin; GLU, glucose; HCO3-, bicarbonate; IRON, iron; K, potassium; MG, magnesium; NA, sodium; PHOS, phosphorus; T-BIL, total bilirubin; TP, total protein; (H), high; (L), low; RI, reference interval.

**Table 2:** Venous blood gas panels in chronological order of a 3-year-old Posavac Hound with pyogranulomatous immune-mediated thyroiditis.

Test	Day 1	Day 2	Day 3	Day 4	Day 7	Day 64	RI	Units
pH	7.228 (L)	7.255 (L)	7.41	7.394	7.397	7.41	7.33–7.45	—
pvCO2	54.0 (H)	40.6 (H)	57.4 (H)	42.1 (H)	42.8 (H)	43.6 (H)	24–39	mmHg
pvO2	28.3 P	70.6	34.8	39.7 (L)	46.4 (L)	39.6 (L)	67–92	mmHg
HCO3	21.7	17.4 (L)	22.9	22.3	25.3	26.6	17–27	mEq/L
ABE	−6.4	−9	−5.3	−1.7	0.9	2.1	−3 to 3	mmol/L
Na	150	146	143	147	146	146	145–156	mEq/L
K	4.5	3.9 (L)	4.8	4.3	4.7	3.7 (L)	4.1–5.6	mEq/L
Cl	121 (H)	120 (H)	113	111	109	115 (H)	104–113	mEq/L
AG	12.4 (L)	12.7 (L)	10.5	14.5	14.3	14.6	13–24	mEq/L
iCa	1.54 (H)	1.49 (H)	1.43 (H)	1.37	1.36	1.29	1.12–1.40	mmol/L
GLU	123 (H)	118 (H)	102	124 (H)	100	123 (H)	70–141	mg/dL
LAC	1.1	0.9	1	1.6 (H)	1.5 (H)	3.3 (H)	0.2–1.44	mmol/L
HGB	16.3	15.7	14.9	14	13.8	18.3	13.0–20.0	g/dL
CREA	1.08	0.83	0.95	1.04	1.03	0.72	0.4–1.9	mg/dL

pH, blood pH; pvCO_2_, venous partial pressure of carbon dioxide; pvO_2_, venous partial pressure of oxygen; HCO_3_, bicarbonate; ABE, actual base excess; Na, sodium; K, potassium; Cl, chloride; AG, anion gap; iCa, ionized calcium; GLU, glucose; LAC, lactate; HGB, hemoglobin; CREA, creatinine; (H), high (above reference range); (L), low (below reference range); RI, reference interval.

On day 2 of hospitalization, the dog’s condition acutely deteriorated. He became obtunded with progressive cervical swelling, severe hypothermia (33.9 °C/93.1 °F), severe sinus bradycardia (50 bpm), and hypoventilation (12 brpm). A single dose of dexamethasone sodium phosphate (dexSP; 0.1 mg/kg IV) was administered. Directly following this injection, the dog’s vital signs, mentation, and neck swelling subjectively improved.

On day 3, the dog underwent a whole-body CT angiogram. Imaging confirmed marked bilateral thyroid enlargement (~4 cm) with central cavitations, and similar cavitated nodules were present in adjacent ventral cervical soft tissues (Supplementary Figure 2). On unenhanced CT, the thyroid lobes appear diffusely hypoattenuating (32–45 HU), in contrast to the 80–100 HU typical of iodine-rich normal thyroid tissue (20), and comparable to masseter muscle. This pattern, combined with lobar enlargement, was consistent with thyroiditis. Post-contrast images showed rim enhancement of the lobes with central low-attenuating cores (2.5–20 HU), indicating necrosis or abscessation. Thickened peri-glandular tissues with contrast uptake and fat stranding indicated regional cellulitis and steatitis. Additional findings were bilateral mandibular salivary gland enlargement with small hypoattenuating foci (≤0.4 cm), severe bilateral medial retropharyngeal lymphadenopathy with perinodal stranding and central low attenuation, and rim-enhancing cavitated nodules in the sternothyroideus and sternohyoideus muscles.

Multiple cavitated nodules and masses were found within the lungs (Supplementary Figure 3), with the largest measuring 4.6 cm in the left caudal lobe. The spleen also exhibited heterogeneous enhancement with numerous hypoattenuating, wedge-shaped regions. Additional findings included bilateral mandibular salivary gland enlargement and severe facial and ventral cervical edema. Repeat FNAs of the pulmonary, splenic, and thyroid masses were non-diagnostic. A surgical biopsy of the thyroid region yielded only adipose tissue with steatitis. Following the CT, dexSP (0.1 mg/kg IV q24h) was reinitiated, and levothyroxine (0.013 mg/kg PO q12h) was started. Following the re-initiation of this combined therapy, the dog’s clinical signs, appetite, and demeanor rapidly improved.

On day 7, he was discharged on levothyroxine (0.013 mg/kg PO q12h) and prednisolone (~1 mg/kg PO q12h). An expanded serum thyroid panel, submitted pre-treatment, returned confirming severe primary hypothyroidism: markedly elevated TSH, undetectable TT4, diminished free thyroxine (FT4), and markedly elevated thyroglobulin autoantibodies (TgAA) (Table 3).

**Table 3:** Thyroid hormone concentrations of a 3-year-old Posavac Hound with steroid-responsive subacute pyogranulomatous thyroiditis^a^.

Test	Day 1^b^	Day 1	Day 64	Day 120	Day 235	RI	Units
TT4	<0.5 (L)	0 (L)	37	27	37	9–52	nmol/L
TT3	—	0 (L)	1.1	1	0.7	0.8–2.1	nmol/L
FT4 by CLIA^b^	<3.86 (L)	—	—	—	—	9–32	pmol/L
FT4 by RIA	<3.86 (L)	1 (L)	29	26	26	9–39	pmol/L
TSH	5.54 (H)	5.39 (H)	0.02	0.02	0.02	0–0.58	ng/mL
TgAA	—	131 (H)	3	0	0	0–35	%
Specific Binding TgAA	—	127 (H)	—	—	—	< 10	%
T4AA	—	5	6	5	7	0–20	%
T3AA	—	8	2	3	5	0–10	%
Ionized Calcium	1.55 (H)	1.34	—	—	—	1.25–1.45	mmol/L
PTH	—	3.2	—	—	—	1.10–10.6	pmol/L
PTH-rP	—	0	—	—	—	0–1.0	pmol/L

a All results were performed by the Michigan State University Veterinary Endocrinology Diagnostic Laboratory, unless otherwise indicated.

b Indicates that the tests were performed, and the results were attained at Colorado State University Veterinary Diagnostic Lab.

CLIA, chemiluminescent immunoassay; FT4, free thyroxine; H, high; L, low; PTH, parathyroid hormone; PTH-rP, parathyroid hormone-related protein; RIA, radioimmunoassay; RI, reference interval; T3AA, T3 autoantibody; TgAA, thyroglobulin autoantibody; TT3, total triiodothyronine; TT4, total thyroxine; T4AA, T4 autoantibody; TSH, thyroid-stimulating hormone.

Follow-up CT scans at 31 and 64 days showed a favorable response. The thyroid lobes became atrophied, the pulmonary lesions resolved, and the lymphadenopathy subsided (Supplementary Figure 4). A repeat expanded thyroid panel at 64 days showed normalization of all thyroid values (Table 3). Based on clinical response and laboratory trends, prednisolone was maintained at ~1.2 mg/kg/d for 3 months, followed by a ~ 50% dose reduction every 3 months until discontinuing. Levothyroxine was continued long-term and subsequently increased (0.023 mg/kg PO q12h) in light of the dog’s recovery and presumed normalization of metabolic clearance. At the time of the last follow-up (day 235), the dog remained clinically normal with no recurrence of cervical swelling or pulmonary signs.

Given the suspicion of an immune-mediated etiology, serum protein electrophoresis and immunofixation were performed on serum samples from two time points (Supplementary Figure 5): the initial sample (day 0), collected prior to any steroid administration, and a sample from hospitalization on day 4 following a low-dose steroid injection. On day 0, the sample revealed marked hypoalbuminemia (1.84 g/dL; RI: 2.77–3.74 g/dL) and a modest increase in the beta-1 globulin fraction (1.06 g/dL; RI: 0.37–0.81), with subjectively corresponding increased IgG4 labeling on immunofixation. On day 4, the sample showed partial improvement in the beta-1 globulin fraction (0.87 g/dL; RI: 0.37–0.81) but a paradoxical subjective increase in IgG4 labeling. These findings were interpreted as evidence of an IgG4-associated immune response and suggested that a single low-dose steroid was insufficient to suppress the underlying inflammation. This interpretation was supported by the dog’s subsequent clinical improvement following the initiation of a high-dose and sustained steroid taper regimen.

## Discussion

The term steroid-responsive immune-mediated thyroiditis (SRIMT) is proposed to distinguish this abrupt, fulminant presentation from both chronic lymphocytic thyroiditis and infectious thyroid disease in dogs. This dog developed sudden bilateral cervical swelling with stridor and dysphonia, as well as cavitated, hypoattenuating thyroid lobes and putatively sterile pyogranulomatous lesions in the lungs and lymph nodes (Supplementary Figures 1, 2). These features mirror the rapid clinical course of acute or subacute thyroiditis in people (3, 4, 6), yet with more extensive systemic involvement. Endocrine testing confirmed severe primary hypothyroidism, with total and free thyroxine below assay detection and markedly elevated thyroid-stimulating hormone (Table 3). The combination of acute onset, characteristic imaging finding, sterile multi-organ inflammation, and dramatic response to immunomodulatory steroid therapy does not fit any previously recognized canine thyroid disorder, supporting SRIMT as a provisional, distinct phenotype and arguing for further characterization of the pathogenesis and clinical progression of acute SRIMTs in dogs.

In people, subacute (de Quervain’s) thyroiditis typically presents with painful thyroid swelling and a transient thyrotoxic phase without multisystem lesions (6, 21). Hashimoto’s thyroiditis evolves gradually without acute systemic inflammation, and Riedel’s thyroiditis produces dense fibrosis rather than cavitation or rapid clinical progression (6, 22). Chronic lymphocytic thyroiditis in dogs progresses insidiously to atrophy without systemic pyogranulomatous inflammation (17). In contrast, this dog’s increased serum IgG4 fraction, a marker of human IgG4-related disease (23), indicated a potential parallel mechanism for this inflammatory syndrome. While this finding is not diagnostic, it highlights IgG4-driven autoimmunity as a potential pathogenic mechanism in dogs that warrants further investigation. This contrasts sharply with typical canine lymphocytic thyroiditis, which, unlike its human Hashimoto’s counterpart, rarely involves systemic inflammation or the aggressive, fibrotic, or necrotizing features seen in human Riedel’s or de Quervain’s thyroiditis, or in this case (23).

Although a deep cervical infection was initially suspected, extensive microbiologic workup, including cultures, PCR assays, and serology, was uniformly negative. The patient was on broad-spectrum antibiotics for 4 days and experienced no improvement during this time. Contrastingly, the cervical mass began to decrease within 8 h of dexamethasone administration, which occurred before any additional antibiotic dose. Ionized calcium normalized with steroid administration (Table 4), albumin improved by day 31 (Table 1), and thyroglobulin autoantibodies returned to baseline by day 64 (Table 3). Moreover, the lung nodules and splenic foci also regressed on steroid therapy but did not appear antibiotic-responsive.

**Table 4:** Complete blood count panels in chronological order of a 3-year-old Posavac Hound with pyogranulomatous immune-mediated thyroiditis.

Test	Day 1	Day 4	Day 31	Day 235	RI	Units
PP	6.5	8.1 (H)	7.7 (H)	6.4	6.0–7.5	g/dL
RBC	6.69	5.55	6.34	6.91	5.5–8.5	10^6^/μL
HCT	48	39 (L)	49	53	40–55	%
HGB	17.1	14	16.9	17.3	13.0–20.0	g/dL
MCV	72	70	77 (H)	76 (H)	62–74	fl
MCHC	35	36	35	33	33–36	g/dL
RDW	12.6	12.3	14.7 (H)	13	12–15	%
ABS. RETIC	51.1	17.3	180.3 (H)	40.5	0–100.0	10^3^/μL
WBC	24.2 (H)	26.8 (H)	16.1 (H)	12.4	4.5–15.0	10^3^/μL
NEUT	20.0 (H)	23.6 (H)	14	10.7	2.6–11.0	10^3^/μL
BAND	—	—	—	1.0 (H)	0–0.2	10^3^/μL
LYMPH	0.7 (L)	0.8 (L)	0.6 (L)	0.1 (L)	1–4.8	10^3^/μL
MONO	2.7 (H)	1.6 (H)	0.5	0.5	0.2–1.0	10^3^/μL
SEG	19.8 (H)	24.4 (H)	15.0 (H)	11.8 (H)	2.6–11	10^3^/μL
MPV	8.6	11.2 (H)	8.5	8.3	7.5–14.6	fl
PLT	398	258	317	238	200–500	10^3^/μL

PP, plasma protein; RBC, red blood cells; HCT, hematocrit; HGB, hemoglobin; MCV, mean corpuscular volume; MCHC, mean corpuscular hemoglobin concentration; RDW, red cell distribution width; ABS. RETIC, absolute reticulocyte count; WBC, white blood cells; NEUT, neutrophils; BAND, band neutrophils; LYMPH, lymphocytes; MONO, monocytes; SEG, segmented neutrophils; MPV, mean platelet volume; PLT, platelets; (H), high (above reference range); (L), low (below reference range); RI, reference interval.

Alternative diagnoses were systematically excluded. Alternative diagnoses were systematically excluded. Deep cervical infection or abscessation was considered unlikely given the lack of response to 4 days of broad-spectrum antibiotics and the rapid improvement following steroid administration. A migrating foreign body typically causes unilateral tract and focal abscess, but not bilateral thyroid necrosis and systemic pulmonary lesions. Neoplastic processes such as thyroid carcinoma or lymphoma, were considered, but the imaging findings (bilateral, cavitated, and non-invasive) and rapid resolution with steroids are not typical for malignancy. Deep sterile panniculitis normally causes diffuse subcutaneous fat inflammation with a waxing and waning course, unlike the rapid, localized changes seen here. Myxedema coma, a consideration for obtundation and bradycardia in severe hypothyroidism, was unlikely given the rapid rebound in mentation, normothermia, and absence of classic skin changes (non-pitting thickening of skin and subcutaneous tissues). Metastatic carcinoma was ruled out due to the absence of invasive margins on CT, the absence of neoplastic cells on cytology, the response to steroids, and clinical progression. Granulomatous infections, including fungal and mycobacterial diseases, would be expected to progress on immunomodulatory steroid therapy or reappear after antibiotics are stopped; negative cultures and persistent lesion quiescence of antimicrobials argue against these possibilities. Foreign-body reactions are typically unilateral and associated with identifiable material; none of these features were present. Human granulomatous thyroiditis may respond to steroids but lack widespread systemic pyogranulomas and necrotizing lesions. The systemic, multi-organ pyogranulomatous inflammation, rapid steroid response, and absence of characteristic histology effectively rule out classic granulomatous thyroiditis in this case.

The main limitation remains the absence of thyroid histopathology, since cavitation and necrosis precluded follicle sampling. Nevertheless, CT initially showed distorted yet identifiable thyroid lobes with diffuse hypoattenuation indicative of inflammatory thyroiditis, and follow-up imaging showed progressive atrophy consistent with immune-mediated destruction. That pattern implies that some residual functional tissue is present. Documentation of histologic changes in the thyroid gland, quantification of the serum IgG4 concentration, investigation for IgG4-positive plasma cells and other mechanisms of inflammation within the tumoral masses, and additional evaluation for the inciting cause of the response would strengthen the description of this case, but were not possible. Evaluation of these facets in future cases with a similar presentation is recommended to confirm SRIMT as a distinct entity and characterize the involved pathology. Furthermore, as this is a single case report, the true incidence of this condition cannot be determined, and the lack of long-term evaluation means the ultimate clinical course and prognosis are unknown. Conducting multi-center studies to establish relevant databases for a case series would be invaluable for confirming SRIMT as a distinct entity, establishing its prevalence, and fully characterizing the involved pathology. Nonetheless, the abrupt clinical onset, cavitated thyroid lesions, systemic sterile pyogranulomas, marked thyroglobulin autoantibody titers, serum IgG4 elevations, and sustained glucocorticoid response together support an immune-mediated origin. The sustained normalization of thyroid hormones, resolution of cervical swelling, and regression of pulmonary lesions under combined levothyroxine and prednisolone therapy closely parallel the outcomes reported in human acute thyroiditis (4, 21, 24).

## Conclusion

This report characterizes acute, steroid-responsive, immune-mediated thyroiditis in a dog that has not been previously described in veterinary medicine. The combination of abrupt, non-painful cervical masses with upper-airway noise, profound hypothyroidism, and sterile pyogranulomatous lesions in the lungs, lymph nodes, and perithyroidal tissues parallels the fulminant presentations seen in human acute or subacute thyroiditis. Diagnosis was established by CT demonstrating cavitated thyroid lobes, comprehensive endocrine testing, and IgG4-enriched serum protein electrophoresis and further supported by rapid, sustained remission with prednisone and levothyroxine. At the 7-month follow-up, all systemic lesions had resolved, and thyroid function remained stable, underscoring the effectiveness of combining immunomodulatory steroid therapy with hormone replacement. Although histologic confirmation was not obtained, the convergence of clinical, imaging, serologic, and therapeutic findings supports an immune-mediated origin. Clinicians should include SRIMT in the differential diagnosis of dogs presenting with acute cervical swelling and multi-organ inflammation. Further evaluation of this mechanism of thyroiditis in dogs is warranted.

## Data Availability

The raw data supporting the conclusions of this article will be made available by the authors, without undue reservation.
