# Supplementary material for: Case Report: Steroid-responsive immune-mediated thyroiditis in a young dog with multi-systemic pyogranulomatous inflammation
Source: Front Vet Sci. 2025 Dec 15;12:1662178. doi: 10.3389/fvets.2025.1662178 (PMC12745206; doi:10.3389/fvets.2025.1662178)
Supplement: Supplementary file 1 [file Data_Sheet_1.PDF]

Supplemental Figure 1. Cervical and thoracic ultrasonography demonstrate bilateral thyroid enlargement with multifocal lymphadenopathy and overlying soft tissue edema.

High-resolution ultrasonography was performed using longitudinal, sagittal, and transverse imaging planes with color Doppler to evaluate thyroid morphology and regional lymph nodes. (A) Transverse view of the left thyroid lobe showing moderate parenchymal heterogeneity and mildly increased echogenicity with preserved intraparenchymal vascular flow. (B) Sagittal view demonstrating a well-defined delineation of the left thyroid lobe measuring 2.30 cm in maximal length. (C) Transverse view depicting bilaterally enlarged thyroid lobes with regionally hyperechoic fat and fluid compatible with subcutaneous edema and cellulitis.

Supplemental Figure 2. Select CT images from a 3-year-old Posavec hound with acute immune-mediated thyroiditis. (A) Unenhanced transverse, (B) contrast-enhanced transverse, and (C) contrast enhanced dorsal CT images. In the unenhanced transverse image (A), the thyroid gland exhibits diffuse hypoattenuation (yellow arrows) with a centrally located fluid-attenuating region. In the contrast-enhanced transverse (B) and dorsal (C) images, fluid attenuating areas ranging from 2.5 to 20 HU is observed within the thyroid lobes (yellow asterisks) and retropharyngeal lymph node (green asterisk). This fluid attenuating area is surrounded by a thick, contrast-enhancing rim. Prominent peri-glandular contrast enhancement and marked fat stranding are also visible, consistent with regional cellulitis and steatitis. *R = Right*

Supplemental Figure 3. Select CT images of multifocal pulmonary lesions in a 3-year-old Posavec hound with acute immune-mediated thyroiditis on day four of hospitalization. The transverse soft tissue post-contrast image (A) shows cavitated, heterogeneous contrast enhancing pulmonary masses in the right and left caudal lung lobes (yellow arrows), with the largest lesion in the right middle lung lobe (3.1cm) featuring a fluid-attenuating core and air bronchograms. The transverse lung window (B) reveals surrounding patchy ground glass opacities, indicative of parenchymal inflammation (yellow dashed arrows). Dorsal post-contrast images (C, D) display additional cavitated lesions with peripheral rim enhancement and internal fluid components, with the largest mass located in the left caudal lung lobe (4.6cm) (yellow arrows). Mild cranioventral pleural effusion and thickening of the ventral mediastinal pleura are also observed. *R = Right*

Supplemental Figure 4. Select CT images from a 3-year-old Posavec hound approximately 60 days post-treatment of acute immune-mediated thyroiditis with steroid therapy and thyroid supplementation. Transverse lung window CT (A, B) and dorsal lung window (D) show significant reduction in the right and left caudal lung lobe masses, with the right caudal lung lobe nodule decreasing from 2.1 cm to 1.4 cm, and the left caudal lung lobe nodule reducing from 1.8 cm to 9.5 mm. Central gas cavitation is visible in the right caudal lobe, indicative of pneumatocele formation (yellow arrows). The previously diffuse nodular pulmonary pattern has improved, with remaining minimal interstitial changes (yellow circle). Dorsal lung window post-contrast images (C) highlight regression of masses and minimal consolidation in the left lung lobes (between yellow arrow heads). Dorsal soft tissue post-contrast (E) reveals marked thyroid lobe atrophy and resolution of prior cervical lymphadenopathy (yellow brackets). *R = Right*

Supplemental Figure 5. Serum protein electrophoresis and immunofixation before and after treatment.

(A, B) Pre-treatment agarose gel-based serum protein electrophoresis (A) demonstrated hypoalbuminemia and a modest increase in the beta-1 region, which was further characterized on immunofixation (B) as enriched in IgG4. The restricted band in the beta region (LC and IgG4 lanes) suggests a polyclonal but IgG4-dominant response. (C, D) Post-treatment electrophoresis (C) and immunofixation (D) at day 30 showed normalization of albumin concentration, reduced beta-1 region prominence, and a more diffuse IgG4 band, indicating resolution of the acute IgG4-dominant inflammatory response. *WB = whole serum; LC = light chain.*
