# Supplementary figures and images for: Case Report: Steroid-responsive immune-mediated thyroiditis in a young dog with multi-systemic pyogranulomatous inflammation
Source: Front Vet Sci. 2025 Dec 15;12:1662178. doi: 10.3389/fvets.2025.1662178 (PMC12745206; doi:10.3389/fvets.2025.1662178)

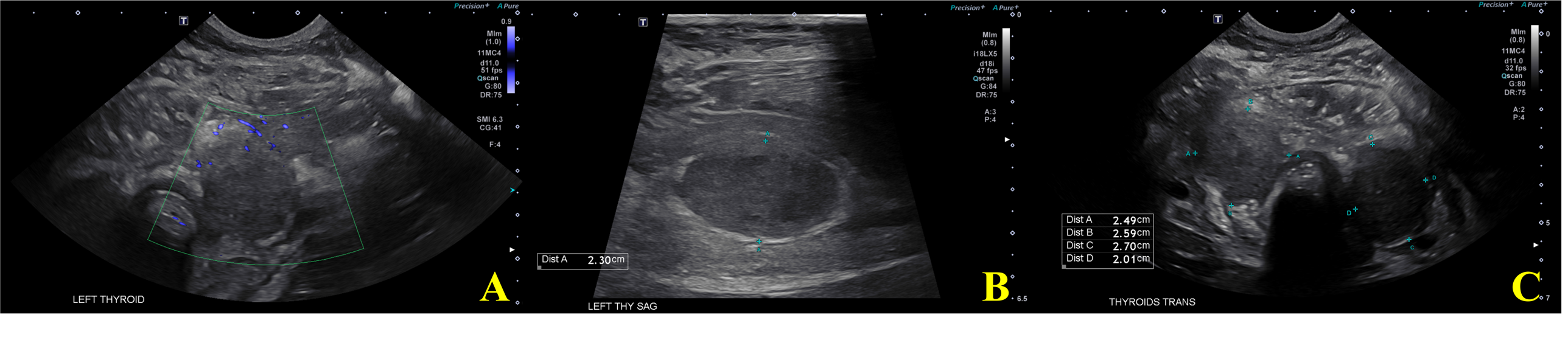

Supplement: Supplementary file 2 [file Image_1.TIFF]

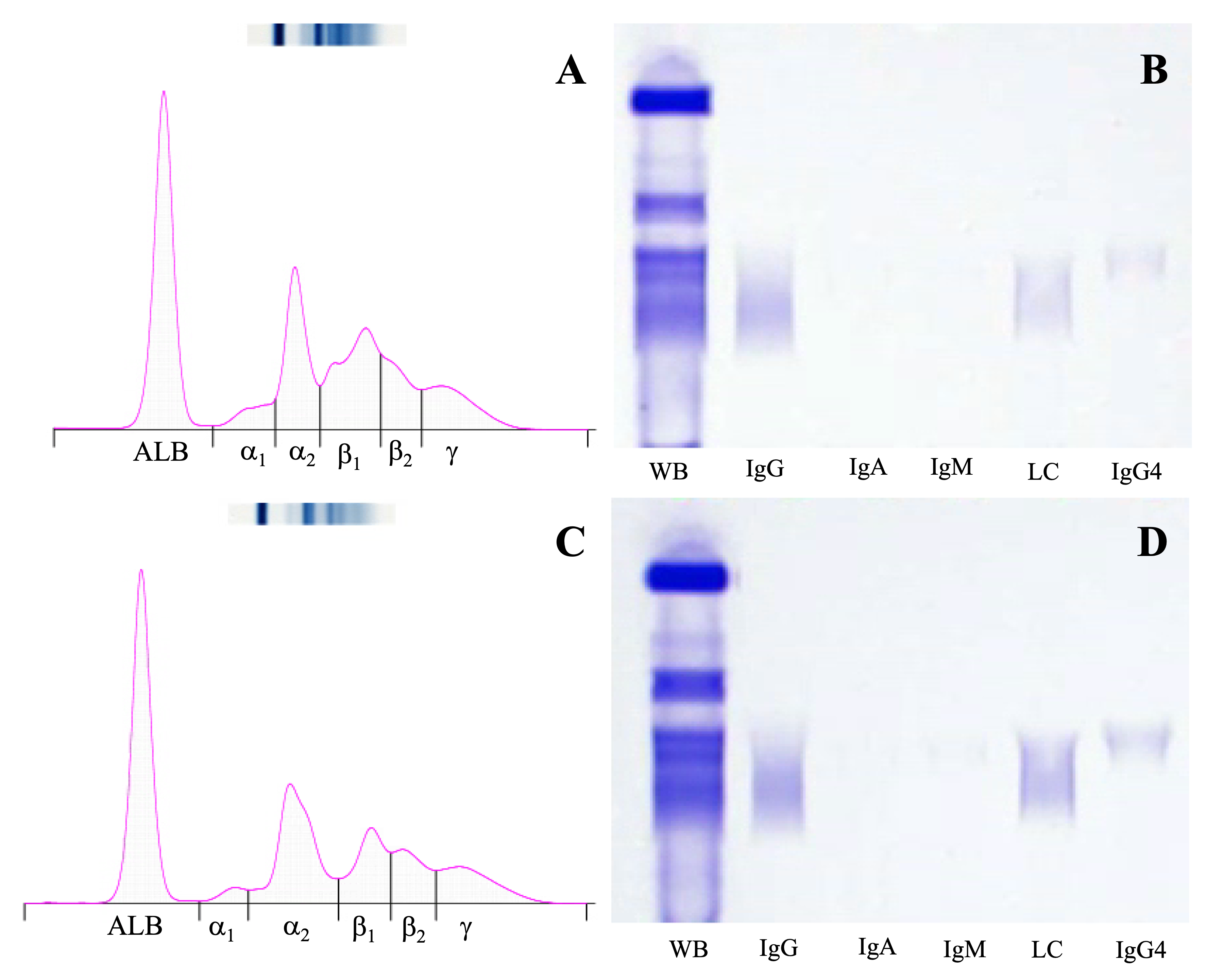

Supplement: Supplementary file 3 [file Image_2.TIFF]

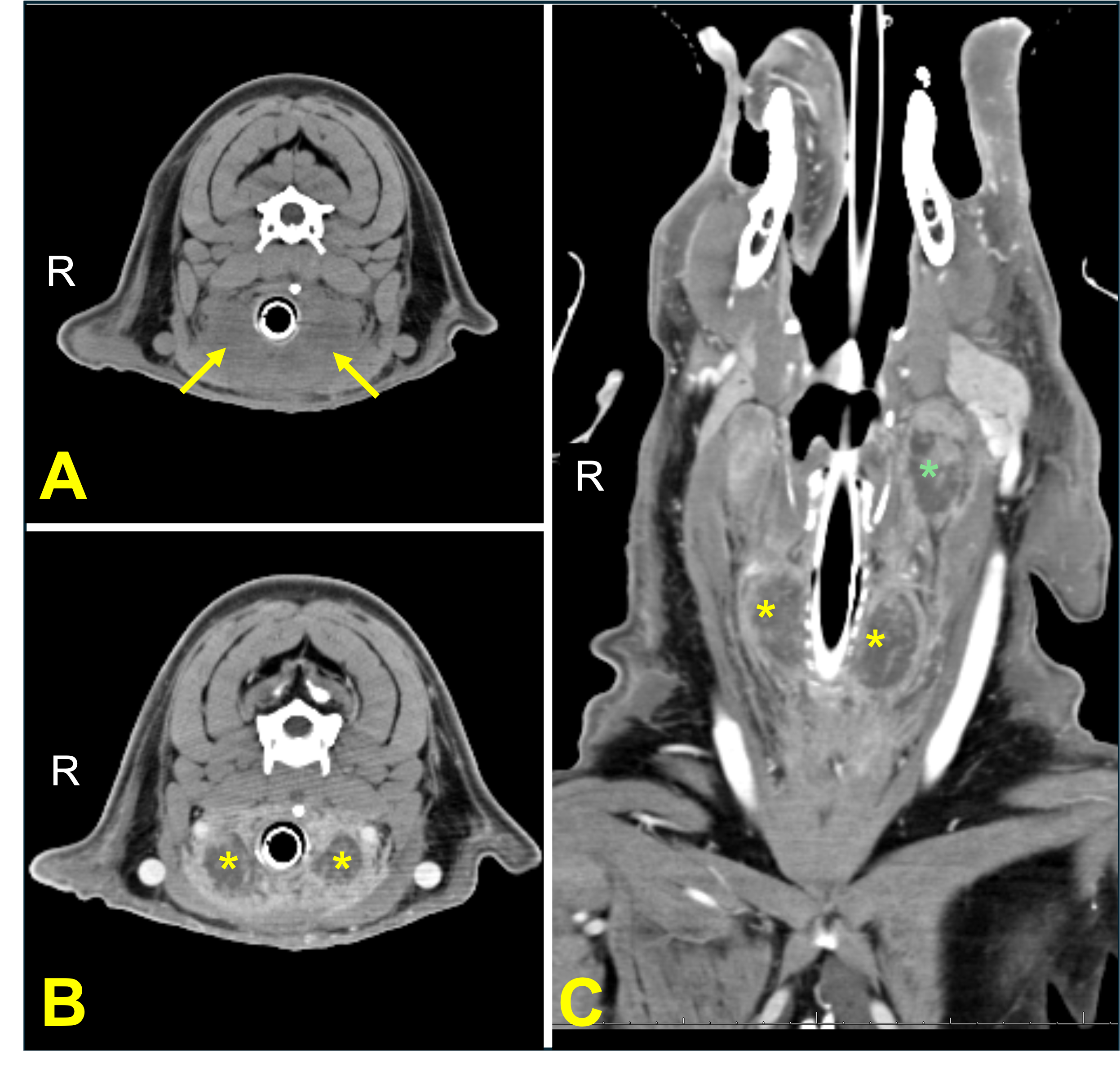

Supplement: Supplementary file 4 [file Image_3.TIFF]

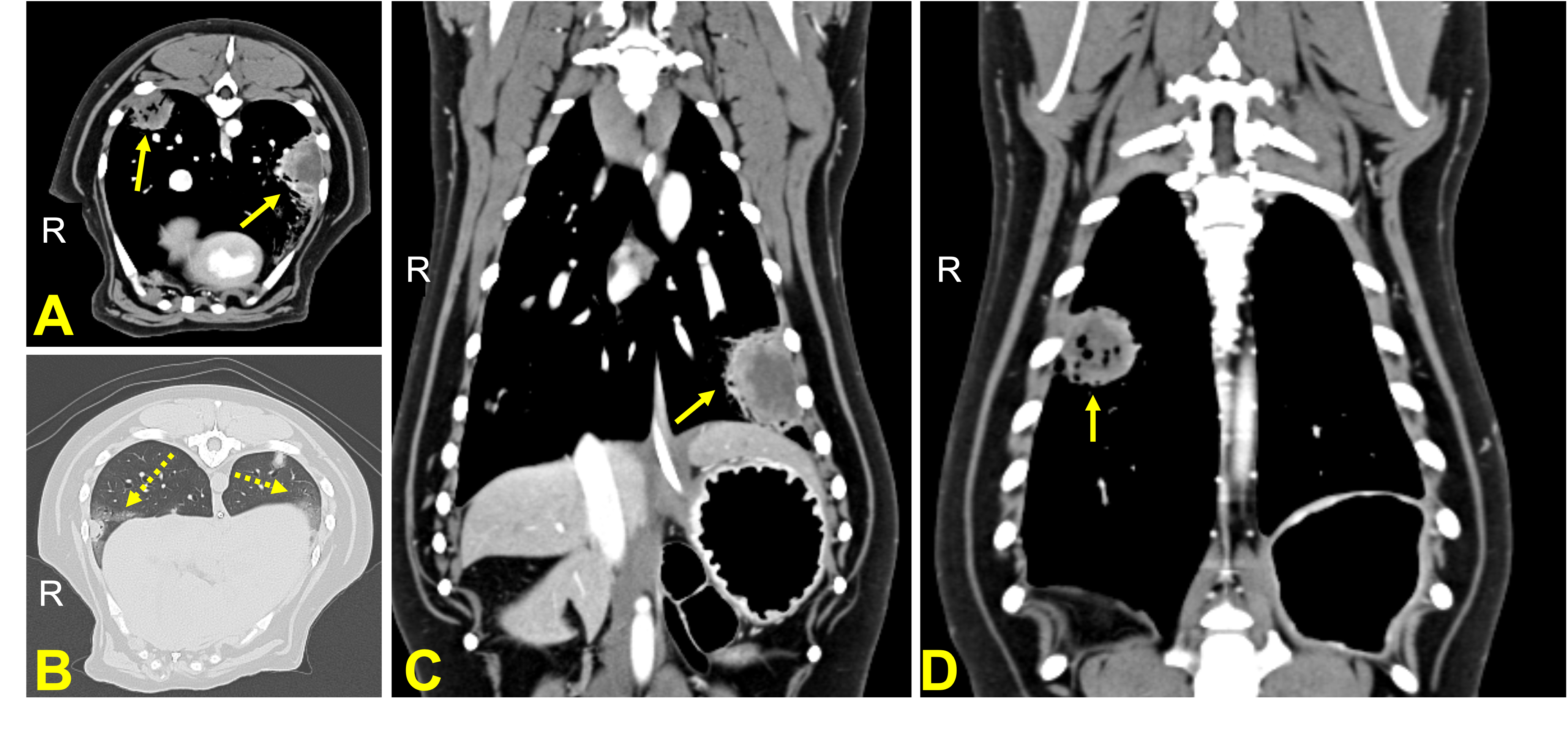

Supplement: Supplementary file 5 [file Image_4.TIFF]

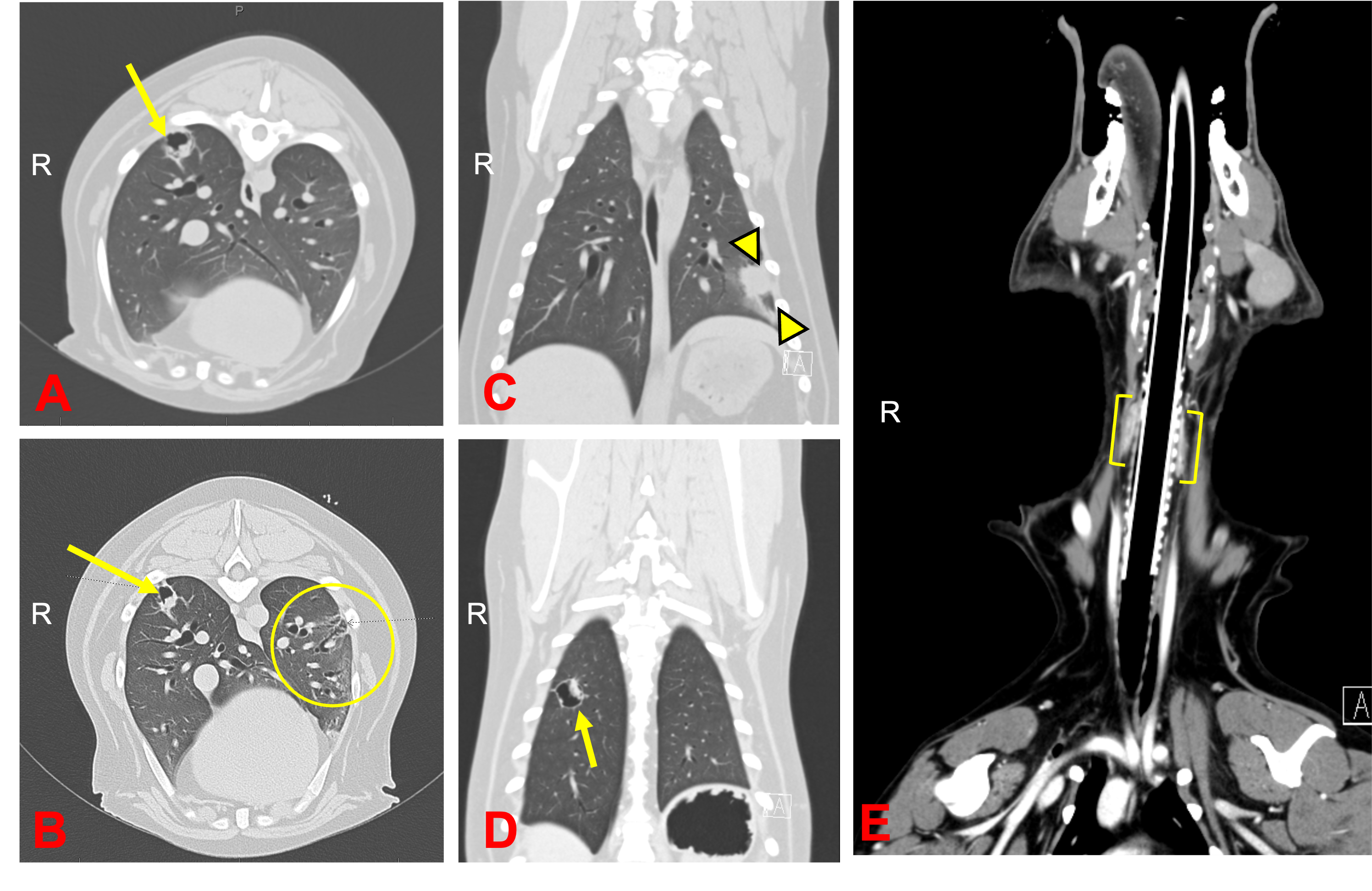

Supplement: Supplementary file 6 [file Image_5.TIFF]
